# Catheter-directed thrombolysis versus systemic anticoagulation in the treatment of symptomatic splanchnic venous thrombosis secondary to acute pancreatitis: a retrospective cohort study

**DOI:** 10.1186/s12893-023-02046-y

**Published:** 2023-07-12

**Authors:** Zongwen Zhang, Lin Gao, Zirui Liu, Gang Li, Bo Ye, Jing Zhou, Lu Ke, Zhihui Tong, Weiqin Li

**Affiliations:** 1 Center of Severe Acute Pancreatitis (CSAP), Department of Critical Care Medicine, Jinling Hospital, Affiliated Hospital of Medical School, Nanjing University, Nanjing, Jiangsu China; 2 Center of Severe Acute Pancreatitis (CSAP), Department of Critical Care Medicine, Jinling Hospital, Nanjing Medical University, Nanjing, Jiangsu China; 3 National Institute of Healthcare Data Science, Nanjing University, Nanjing, Jiangsu China

**Keywords:** Acute pancreatitis, Splanchnic venous thrombosis, Catheter-directed thrombolysis, Systemic anticoagulation therapy, Vascular recanalization

## Abstract

**Background:**

Catheter-directed thrombolysis (CDT) has been an important therapy and seems effective in patients with splanchnic venous thrombosis (SVT) secondary to some diseases, but this intervention hasn’t been formally evaluated in the setting of acute pancreatitis (AP).

**Methods:**

This was a retrospective study enrolled patients between January 2013 and December 2018. AP patients who developed SVT-induced symptoms, including intractable ascites and/or enteral nutrition intolerance, were included. Demographics, SVT associated parameters, clinical features and outcomes, long-term quality of life evaluated by using SF-36 questionnaire were compared between CDT group and systemic anticoagulation (SAC) group.

**Results:**

6 patients underwent CDT and 17 received SAC. Patients in CDT group had a higher recanalization rate (100% versus 35.3%; *p* = 0.014) and shorter time to symptoms resolution (median 8 days versus. 31.5 days, *p* = 0.004). Mortality and length of hospital stay were comparable between two groups. The association analysis indicated that CDT use exerted a significantly beneficial effect on recanalization rate (risk ratio, 2.833; 95% CI, 1.489 to 5.393; *p* = 0.002) and time to symptoms resolution (mean difference, -33.333; 95% CI, -64.612 to -2.055; *p* = 0.038). No SVT-related symptoms recurrence was recorded in survivors at six-month follow-up. There was no statistical difference in either item of SF-36 questionnaire between two groups.

**Conclusions:**

Compared with SAC, CDT may facilitate vascular recanalization and shorten symptom resolution for symptomatic SVT.

## Introduction

Splanchnic venous thrombosis (SVT), a peripancreatic vascular complication secondary to various diseases, including acute pancreatitis (AP) and chronic pancreatitis, may involve one or more veins, such as portal vein (PV), splenic vein (SPLV) and superior mesenteric vein (SMV). [1] The incidence of SVT varies widely, ranging from 1.8 to 36.5%, due to the heterogeneity of the study subjects (acute versus chronic pancreatitis, mild versus severe AP) [2–5].

The development of SVT may cause serious clinical consequences, such as persistent abdominal distention, intractable ascites, enteral nutrition intolerance, even upper gastrointestinal bleeding, small bowel ischemia and hepatic failure [6]. Although limited literature reported that the incidence of these potentially fatal consequences was relatively low [7], they were associated with unfavorable clinical outcomes, including increased mortality, prolonged intensive care unit (ICU) and hospital stays, and impaired quality of life [8, 9]. Therefore, this subset of patients may merit therapeutic interventions.

It was recommended that systemic anticoagulation (SAC) should be used in patients with symptomatic SVT and without bleeding risk or other contraindications [10]. Some recent studies demonstrated that the benefits of SAC outweigh the bleeding risks, especially in patients with SVT-induced symptoms [11, 12]. However, SAC may last for several months and require regular hospital visits and coagulation assessment after hospital discharge, which was costly and time-consuming. Catheter-directed thrombolysis (CDT), which enables delivering thrombolytic drugs to local blood vessels via a transjugular or transhepatic route, is an alternative. It has been applied in some patients with SVT secondary to cirrhosis with or without Factor V Leiden heterozygote, post hepatic transplants deep vein thrombosis and pulmonary embolism, etc. Several studies have reported that these attempts are effective in thrombus resolution and recanalization [13–17]. However, to date, no study has reported the feasibility of CDT in AP-associated SVT.

Hence, this study aimed to evaluate the safety and efficacy of CDT in AP patients with symptomatic SVT, by comparing the clinical outcomes with those of patients undergoing therapeutic SAC.

## Methods

### Study design

This was a retrospective cohort study conducted at Center of Severe Acute Pancreatitis of Jinling Hospital, Nanjing, China. All the data were retrospectively extracted from a web-based electronic database (Acute Pancreatitis Database) with the approval of institutional review board (2018JLAPDMC-011). Broad informed consent was obtained from each participant on academic use of the clinical and laboratory data during hospitalization. The study was carried out in accordance with current revision of the 1964 Helsinki Declaration and with the laws and regulations of the nation.

### Patients and definitions

From January 2013 to December 2018, patients with a diagnosis of SVT secondary to AP were retrospectively screened for potential inclusion. Patients with low risk of bleeding who developed SVT-induced symptoms, including intractable ascites or enteral nutrition intolerance, were included in this study. Patients who met the following criteria were excluded: (1) younger than 18 years old or older than 80 years old, (2) with a history of chronic pancreatitis, known malignancy, cirrhosis, or established portal hypertension, (3) had a high risk of bleeding, since anticoagulation therapy was contradicted in these patients.

The severity of AP was classified in accordance to the Revised Atlanta Classification (RAC) definitions [18]. Based on computed tomography venography (CTV) or contrast-enhanced CT (CECT) imaging, the diagnosis of SVT and recanalization of veins were determined. The recanalization was defined as the complete resolution of thrombus. The radiologists were blinded to the clinical history and disease course of the patients. SVT was diagnosed when a certain thrombus was detected in a vein, or when a vein was compressed or not visualized with the presence of collaterals.

SVT-induced symptoms mainly included intractable ascites and enteral nutrition intolerance. Intractable ascites was defined as presence of more than 1000 ml ascites per day after ruling out other possible causes, such as hypoalbuminemia, chylous ascites, and disease-related ascites in the acute phase. Enteral nutrition intolerance caused by SVT was defined as the appearance of gastrointestinal symptoms, such as abdominal pain, abdominal distention or vomiting due to the use of enteral nutrition after feeding goals had been achieved already for a period of time, and other contributing factors should be ruled out [12]. Resolution of SVT-induced symptoms was defined when daily ascites was less than 100 ml for three consecutive days or re-achieving the enteral nutrition feeding goals. The bleeding in this study referred to massive intra-abdominal bleeding, which was defined as significant hemodynamic deterioration and/or a sharp decrease in hemoglobin concentration of > 2 g/dL, and would need blood transfusion or subsequent intervention for hemostasis [19].

### Treatment and grouping of the patients

Patients were grouped as CDT group or SAC group based on the treatment they received for SVT. The treatment modalities were decided by the multidisciplinary treatment team which contains surgeons, radiologists, intensivists and etc. Patients in the SAC group received a therapeutic dose (1 mg/kg body weight per 12 h) of low molecular weight heparin (LWMH, enoxaparin) by subcutaneous injection.

The CDT procedures were conducted under the guidance of digital subtraction angiography (DSA). Transjugular access was the most commonly used route. Briefly, the right internal jugular vein was punctured percutaneously, and a sheath was advanced into the inferior vena cava. The right hepatic vein was then cannulated with the sheath advanced, then a hepatic venogram was obtained. Under the fluoroscopic guidance, the Rosch-Uchida system needle was used to access the portal venous system. Then the angled glide wire and a 5-Fr multiple-side-hole infusion catheter were then placed into the vein, and catheter-directed thrombolysis was immediately initiated using urokinase and LWMH solution for the following three consecutive days. The decision on whether a vascular stent should be placed was made by the treating clinicians. Urokinase and LWMH were given continuously at a dose of 400,000U/day and 8,200U/day, respectively. The catheter was removed after the disappearance of the thrombus or after 72 h of thrombolytic therapy, whichever came first. Another catheter angiography was performed to evaluate the latest thrombus status after the treatment. If thrombus still existed, thrombolysis would be administrated for another three days.

For other treatments, all the patients were managed by the same team throughout the study period, including fluid resuscitation, percutaneous catheter drainage, nutrition therapy, etc., according to the international guideline [20].

### Data collection

Demographic characteristics and clinical features, including age, gender, etiology, disease severity, acute physiology, and clinical health evaluation score-II (APACHE II) score, and CT severity index (CTSI) at admission, were collected. SVT associated parameters, including the interval from AP onset to the detection of SVT-induced symptoms, the localization of SVT, time to symptoms resolution, symptoms resolution rate, and recanalization rate, were recorded. In addition, clinical outcomes, including hospital mortality, the length of hospital and ICU stay, the incidence of massive intra-abdominal bleeding and CDT-related complications were collected. The efficacy was assessed by time to symptoms resolution, symptoms resolution rate, and recanalization rate, and the safety was assessed by the incidence of bleeding, mortality and CDT-related complications. Moreover, a six-month follow-up was carried out to assess the SVT recurrence and long-term health-related quality of life through SF-36 questionnaire.

### Statistical analysis

The median (interquartile range) was used to describe continuous variables unless mentioned otherwise, whereas frequency and percentage were used to summarize categorical variables. Continuous variables were compared using the Mann-Whitney U test, and categorical data were analyzed with Fisher’s exact test. The association between different treatment modalities and categorical clinical outcomes was evaluated by Modified Poisson regression. Moreover, the risk ratio (RR) and mean difference (MD), together with their 95% confidence interval (CI), were calculated. Statistical analyses were performed using SPSS statistical software (version 24.0, IBM Analytics, Armonk, NY). A two-sided probability (*p* value) of < 0.05 was considered statistically significant.

## Results

### Patient characteristics

**Fig. 1:**
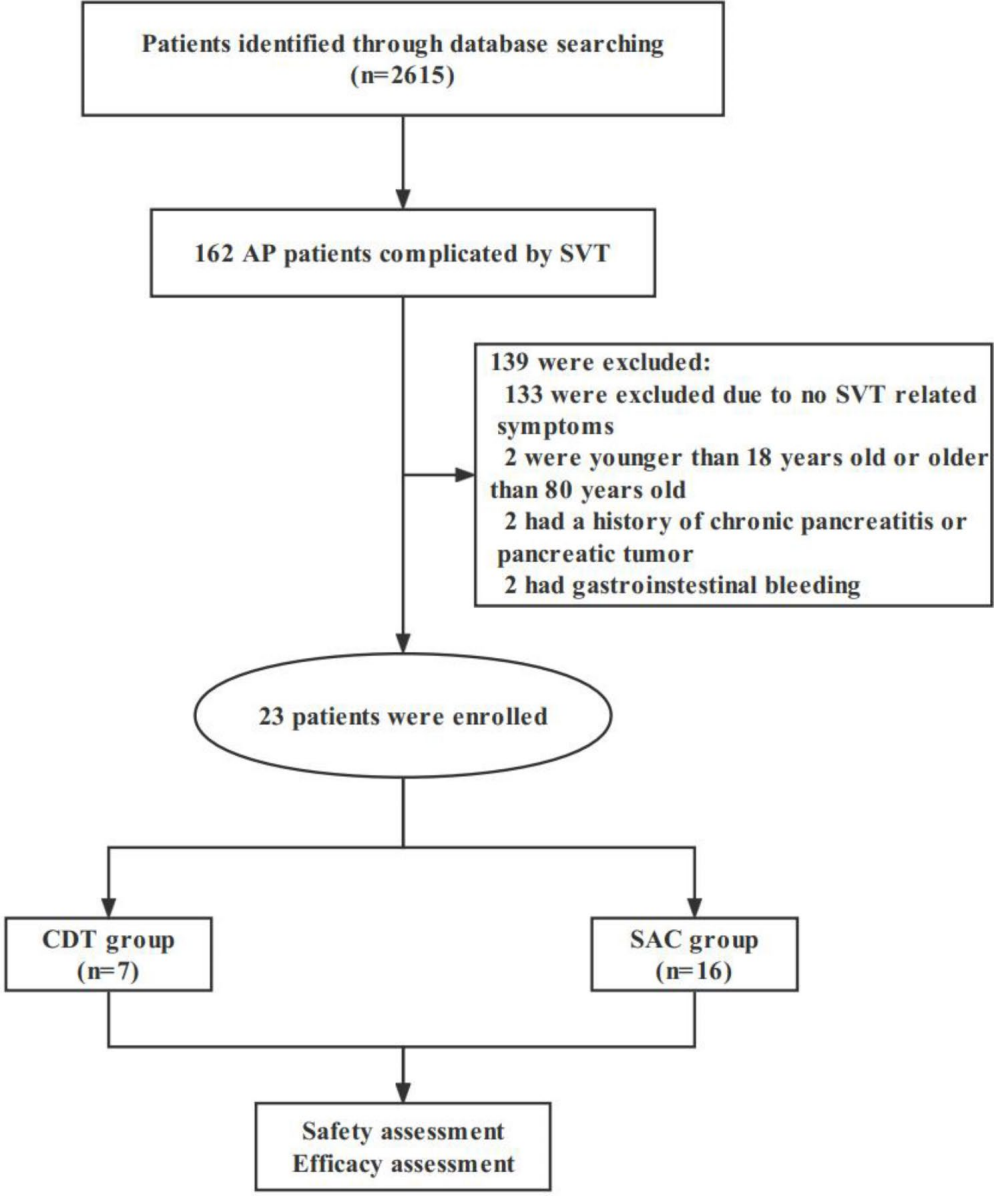
Study flowchart

**Fig. 2:**
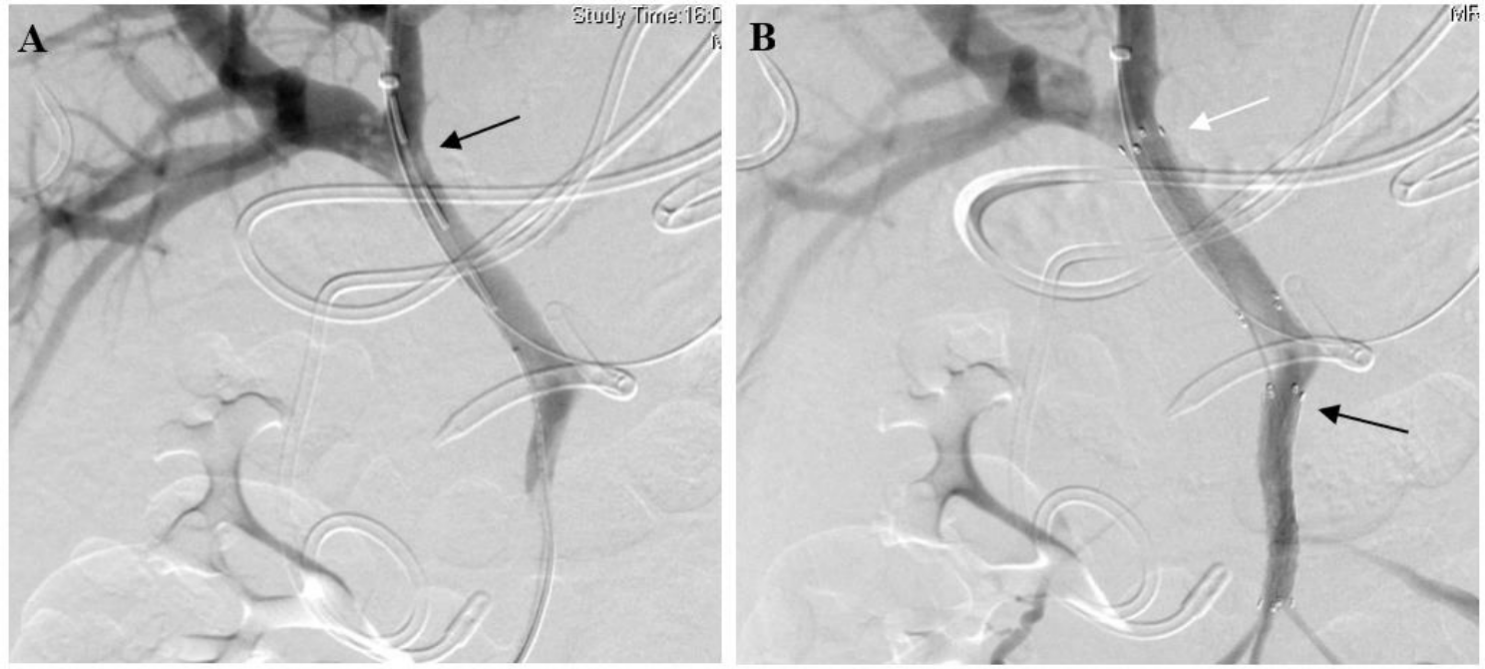
The angiography images of a 49-year-old patient. **A**: the black arrow pointed out thrombus formation in the portal vein; **B**: image after catheter-directed thrombolysis and the black arrow showed that the stent was placed in the vein; the white arrow showed that the thrombus in the portal vein had decreased after thrombolysis

During the six-year period, a total of 2615 patients were admitted with a diagnosis of AP, and 162 (6.2%) were diagnosed with SVT (Fig. 1). Of these patients, 23 (14.2%) developed SVT-induced symptoms, and among them, six patients underwent CDT, while 17 patients received therapeutic SAC. Figure 2 showed the angiography images of a 49-year-old patient before (Fig. 2A**)** and after (Fig. 2B) CDT. Demographics and baseline clinical features of included patients were shown in Table 1. All the study patients were categorized as severe AP. Intractable ascites was the predominant symptom, and was detected in 20/23 (87.0%) patients, followed by enteral nutrition intolerance (7/23, 30.4%). All the 20 patients with intractable ascites underwent abdominal paracentesis.

**Table 1:** Demographics, etiology and disease severity in the study cohort

Variables	CDT group (n = 6)	SAC group (n = 17)	*p* Value	
Gender, M/F	4/2	15/2	0.473	
Age, year	44.5 (39.8–52.8)	52 (43–65)	0.220	
Etiology, n			0.516	
Biliary Hyperlipidemia	5 1	11 6		
SVT-induced symptoms, n				
Intractable ascites	5	15	1.000	
Enteral nutrition intolerance	1	6	0.621	
Interval from AP onset to the detection of symptoms, days	47.5 (32.2–76.2)	54 (47-60.5)	0.861	
CTSI at admission	10 (8–10)	8 (8–10)	0.293	
APACHE II Score at admission	17 (11–19)	12 (7–15)	0.149	

CDT, catheter-directed thrombolysis; SAC, systemic anticoagulation; M, male; F, female; SVT, splanchnic venous thrombosis; APACHE II, Acute Physiology and Chronic Health Evaluation II; CTSI, Computed Tomography Severity Index.

### Pattern of SVT

The localization of SVT was shown in Table 2. Single, double, and triple vessels involvement were seen in five (21.7%), eleven (47.8%), and seven (30.4%) study patients, respectively. SPLV was the most commonly involved vessel (21/23, 91.3%), followed by the SMV in 18/23 (78.3%) patients and PV in 9/23 (39.1%) patients. SPLV was isolated in 4/23 (17.4%) patients while in combination with portal vein and/or superior mesenteric vein in 17/23 (73.9%) patients.

**Table 2:** Localization of splanchnic vein thrombosis

Location of the Thrombus, n	CDT group (n = 6)	SAC group (n = 17)
SMV, n	0	1
SPLV, n	0	4
PV + SPLV, n	1	0
SPLV + SMV, n	3	6
PV + SMV, n	0	1
PV + SMV + SPLV, n	2	5

CDT, catheter-directed thrombolysis; SAC, systemic anticoagulation; PV, portal vein; SMV, superior mesenteric vein; SPLV, splenic vein.

### Clinical outcomes and association analysis

Patients in the CDT group had a higher recanalization rate (100% versus. 35.3%, *p* = 0.014) than patients in the SAC group. Moreover, patients in the CDT group reached symptoms resolution earlier (median 8 days versus 31.5 days, *p* = 0.004), while there was no significant difference in symptoms resolution rates between the two groups. In terms of adverse events, bleeding occurred in 3/6 (50%) patients in the CDT group compared to 7/17 (41.2%) in the SAC group. Moreover, two patients in the CDT group developed post-procedure complications. One had hepatic hematoma who was treated by percutaneous catheter drainage, and the other one had septic shock derived from biliary infection, who was treated by fluid resuscitation and broad-spectrum antibiotics. The clinical conditions of both patients improved after these treatments. No statistical significance was found between two groups, in terms of mortality, length of hospital stay and length of ICU stay (Table 3).

**Table 3:** Recanalization and Clinical Outcomes of the study patients

Variables	CDT group (n = 6)	SAC group (n = 17)	*P* Value
Recanalization, n (%)	6 (100)	6 (35.3)	0.014
Time to symptoms resolution, days	8 (4.25–13.25)	31.5 (29-43.75)	0.004
Symptom resolution, n (%)	4 (66.7)	12 (70.6)	1.000
Length of hospital stay, days	97 (75.2-132.2)	66 (44–97)	0.080
Length of ICU stay, days	80.5 (32.8–95.8)	39 (25-75.5)	0.248
Bleeding, n (%)	3 (50)	7 (41.2)	1.000
Mortality, n (%)	1 (16.7)	6 (35.3)	0.621

CDT, catheter-directed thrombolysis; SAC, systemic anticoagulation; ICU, intensive care unit.

The association analysis indicated that the application of CDT exerted a significantly beneficial effect on the recanalization rate (risk ratio, 2.833; 95% CI, 1.489 to 5.393; *p* = 0.002) and time to symptoms resolution (mean difference, -33.333; 95% CI, -64.612 to -2.055; *p* = 0.038) (Table 4).

**Table 4:** Association analysis for CDT grouping and clinical outcomes

Variables	RR	95%CI		*P* value
		Lower	Upper	
Recanalization	2.833	1.489	5.393	0.002
Symptom resolution	0.944	0.496	1.798	0.862
Bleeding	1.214	0.455	3.240	0.698
Mortality	0.472	0.071	3.162	0.439
	**MD**	**95%CI**		***P*** **value**
		**Lower**	**Upper**	
Time to symptom resolution	-33.333	-64.612	-2.055	0.038
Length of hospital stay	32.235	3.324	67.795	0.073
Length of ICU stay	16.892	-21.636	54.809	0.351

CDT, catheter-directed thrombolysis; RR, risk ratio; CI, confidence interval; MD, mean difference; ICU, intensive care unit.

No SVT-related symptoms recurrence was recorded in survivors in either group at the six-month follow-up. There was no statistical difference in either item of SF-36 questionnaire between the two groups (Table 5).

**Table 5:** The quality of life of survivors evaluated by SF-36 questionnaire at 6-month follow-up

Items	CDT group (n = 5)	SAC group (n = 11)	*P* Value
Physical functioning	75 (67.5–87.5)	80 (70–90)	0.606
Role-physical	0 (0-62.5)	0 (0-100)	0.753
Bodily pain	74 (51.5–74)	70 (51–70)	0.954
General health	70 (55–75)	62 (55–85)	0.909
Vitality	75 (52.5–82.5)	65 (60–85)	0.731
Social functioning	50 (8–10)	62.5 (37.5–87.5)	0.387
Role-emotional	33.3 (0-100)	33.3 (0-100)	0.716
Mental health	80 (54–86)	60 (56–84)	0.531

## Discussion

In this study, CDT grouping was associated with a higher recanalization rate and a shorter time to symptom resolution in AP patients complicated by SVT. However, these benefits failed to translate into improvement in other patient-centered outcomes, such as mortality and length of hospital stay. Moreover, at the six-month follow-up, there was no SVT recurrence and there was no statistical difference in quality of life between two groups.

Consistent with a previous study [7], the results of this study demonstrated that SVT-induced symptoms were infrequent in AP patients. As for asymptomatic SVT, endovascular treatment was unnecessary, and close monitoring of the thrombus status was sufficient [21], while symptomatic SVT may benefit from therapeutic interventions. Therapeutic SAC, the most commonly used intervention for SVT, has been shown safe. In previous studies [11, 22], among patients administered with therapeutic SAC, complications directly attributable to SAC, such as fatal bleeding, were rare. While the benefits of therapeutic SAC were uncertain, for instance, in the study by Gonzelez [23] et al., recanalization was observed in almost a third of patients, irrespective of whether or not they received SAC, let alone the improvements in patient-centered outcomes, such as mortality.

CDT is an invasive intervention for symptomatic SVT. The preference for CDT is based on limited data that suggest similar rates of thrombus lysis compared with SAC and the lower likelihood of bleeding due to the administration of lower doses of thrombolytic agents [24]. Moreover, CDT is commonly considered in the following circumstances [14]. First, patients have thrombosis in multiple splanchnic vessels, such as SMV paired with PV. Second, the symptoms are not improved remarkably or even get worse after systemic anticoagulation. In a study [15] involving 32 patients with acute SMV thrombus who underwent emergency surgery, 17 patients received postoperative SAC, while 15 CDT, and the results showed that CDT was associated with earlier symptoms resolution, shorter length of hospitalization, lower 30-day mortality, and higher 1-year survival rate. In accordance with previous studies, patients undergoing CDT in the current study achieved symptom resolution sooner and had higher rates of SVT recanalization. However, no benefits for survival or length of hospital stay were demonstrated. Unlike SVT induced by cirrhosis or other diseases, AP induced symptomatic SVT could recover with no recurrence when AP and its complications were resolved. Moreover, the quality of life should be paid more attention in a longer term of follow-up in future studies.

A major concern with the use of CDT is the risk of post-procedure complications. The study conducted by Hollingshead et al. [13] showed that the post-procedure complication rate was as high as 60% (12/20), including hematocrit decrease, hematuria, and abdominal bleeding. In this study, two patients (2/6, 33.3%) in the CDT group experienced post-procedure complications, and these complications were managed successfully with conservative treatment in both patients. Hence, the risks and benefits of CDT must be prudently weighed considering the risk of these complications.

The current study may present some limitations. First, the incidence of SVT might be underestimated, as asymptomatic SVT might be missed out because no diagnostic imaging was performed. Second, it was a single-center, retrospective study with small sample size, which may bring bias into the statistical power and interpretation of the results. In addition, this study also had potential selection bias since the decision for CDT or SAC was made mainly by the treating clinician.

## Conclusion

SVT was a common complication in patients with AP, while most patients with SVT would not develop symptoms. Compared with SAC, CDT appeared to be safe and effective in patients with symptomatic SVT secondary to AP. Therefore, more well-designed, prospective, randomized controlled trial were warranted to confirm these findings in future.

## Data Availability

The datasets used and/or analysed during the current study available from the corresponding author on reasonable request.

## List of Abbreviations

AP: Acute pancreatitis
APACHE II: Acute physiology and clinical health evaluation score-II
CDT: Catheter-directed thrombolysis
CECT: Contrast-enhanced CT
CI: Confidence interval
CTSI: CT severity index
CTV: Computed tomography venography
DSA: Digital subtraction angiography
ICU: Intensive care unit
LWMH: Low molecular weight heparin
MD: Mean difference
PV: Portal vein
RAC: Revised Atlanta Classification
RR: Risk ratio
SAC: Systemic anticoagulation
SMV: Superior mesenteric vein
SPLV: Splenic vein
SVT: Splanchnic venous thrombosis
